# Xpert MTB/RIF Ultra Ct Value: A Quick Indicator of Sputum Bacillary Load and Smear Status Prediction in Individuals with Pulmonary Tuberculosis

**DOI:** 10.24248/eahrj.v9i1.823

**Published:** 2025-09-30

**Authors:** Davis J. Kuchaka, Saumu P. Juma, Erick A. Shekimweri, Buliga M. Swema, Philoteus A. Sakasaka, Blandina T. Mmbaga, Margaretha L. Sariko

**Affiliations:** a Kilimanjaro Clinical Research Institute, Moshi, Tanzania; b Kilimanjaro Christian Medical Centre, Moshi, Tanzania; c KCMC University, Moshi, Tanzania

## Abstract

**Background:**

Tuberculosis (TB) remains a global health threat, necessitating continuous advancements in diagnostic techniques for effective management and control. This study aimed to evaluate the diagnostic utility of smear microscopy and the Xpert MTB/RIF Ultra test in TB diagnosis, focusing on the correlation between Cycle threshold (Ct) values and disease severity.

**Methods:**

A prospective cross-sectional study was conducted in the Kilimanjaro region, enrolling 472 participants suspected of pulmonary TB. Sputum samples were subjected to smear microscopy, and Xpert MTB/RIF Ultra testing. Data were analysed using R software. The ROC curve was created to assess the performance of the Ct values, and Spearman's correlation and Mann-Whitney test to evaluate the association of Ct value and smear microscopy. Ethical approval was obtained from local and national ethical review boards.

**Results:**

The study revealed discrepancies between smear microscopy and Xpert MTB/RIF Ultra, testing in identifying patients with high bacterial loads. The Ct value in predicting the smear grading yielded a sensitivity of 71% (95% CI 55.2 – 82.7) and specificity of 79.2% (95% CI 64.1 – 89.2), with an area under the curve (AUC) of 0.806. Analysis of Ct values revealed a negative correlation with smear grading, suggesting the potential utility of Ct values as predictive biomarkers for disease severity.

**Conclusion:**

This study underscores the importance of advanced diagnostic techniques, such as the Xpert MTB/RIF Ultra test, in enhancing TB diagnosis and management. The correlation between Ct values and disease severity highlights the potential of Ct values as predictive indicators, offering promising prospects for personalized treatment strategies. Addressing discrepancies between diagnostic methods and further research into Ct value correlations are essential for refining TB diagnostic protocols and improving patient outcomes globally.

## BACKGROUND

Tuberculosis (TB) remains a significant global health threat, with over 1 million deaths worldwide attributed to the disease annually.^1^ Despite the implementation of effective treatment regimens and national TB control programs, TB persists as a major public health concern. In the absence of proper medical intervention, TB mortality rates can exceed 50%.^2^ The timely and accurate diagnosis of TB is crucial in controlling the pandemic.^3^

Various diagnostic techniques endorsed by the World Health Organization (WHO) have been utilized in TB diagnosis, including smear microscopy (both Ziehl-Neelsen and Auramine methods), culture methods, and more recently, the GeneXpert MTB/RIF Ultra (Xpert MTB/RIF Ultra), MTB/RIF assay, which has been superseded by the MTB/RIF Ultra Cartridge. ^4,1,5^

Smear microscopy for acid-fast bacilli (AFB) is a widely used method for quantifying mycobacterial burden during TB diagnosis.^6^ This technique involves staining sputum samples with a special dye that binds to the mycobacterial cell wall, making the bacteria visible under a microscope. The bacterial load, or “bacillary burden,” in sputum samples indicates the severity of TB infection, as a higher bacterial load typically correlates with more advanced disease and increased infectivity.^7,8^

The Xpert MTB/RIF Ultra test is a molecular diagnostic tool that detects *Mycobacterium tuberculosis* DNA in sputum samples. In this context, the “Ct value” refers to the cycle threshold value, representing the number of amplification cycles needed for the test to detect a positive signal above a certain threshold. A lower Ct value indicates a higher concentration of target DNA in the sample.

The Ct value obtained from the Xpert MTB/RIF Ultra test provides rapid insight into the amount of *M. tuberculosis* DNA in the sputum sample. A lower Ct value suggests a higher bacillary burden, potentially indicating a more severe or advanced stage of pulmonary TB. Given the correlation between bacterial load and smear status, there is interest in exploring whether the Ct value could be a predictive indicator of smear positivity. A lower Ct value may be associated with a higher likelihood of a positive smear result, indicating increased bacterial concentration in the sputum and greater infectiousness.^9^

The Ct value from the Xpert MTB/RIF Ultra test offers valuable information about the severity of pulmonary TB and its potential for transmission. This information can inform clinical decision-making and infection control measures. As medical research and guidelines evolve, it is essential to consult the most current sources for accurate and up-to-date information.

## METHODOLOGY

This prospective cross-sectional diagnostic study was conducted in the Kilimanjaro region, focusing on adults undergoing TB screening. These were patients who had signs and symptoms of TB, including coughing for more than 2 weeks, chest pain, coughing up mucus, unexplained weight loss, night sweats, and fatigue. Patients who were ≥18 years and were able to produce sputum samples. Also, those who consented for the sample to be used for multiple testing were included. The patients who were unable to produce sputum samples and who were already on the treatment were excluded. Participants were consented as they became available. The sample size was calculated using;

n=Z^2^ × p × (1−p)/d^2^ with a prevalence estimate of 12.7% and a margin of error of 3%.

n= (1.96)^2^ × 0.127 × (1−0.127)/(0.03)^2^

n=3.8416 × 0.127 × 0.873/0.0009

n=3.8416 × 0.110871/0.0009

n=0.426045/0.0009≈473.38

n= 473

A total of 472 were enrolled in the study and were considered sufficient for the study.

All patients, irrespective of their TB screening results, were included in the study and underwent comprehensive testing. Samples obtained from participants were subjected to Xpert MTB/RIF Ultra assay and AFB smear microscopy using Auramine staining.

The Xpert MTB/RIF Ultra utilized in this study incorporates two multicopy amplification targets (IS6110 and IS1081) and the RIF resistance-determining region (RRDR) of the rpoB gene. Additionally, the assay includes an internal control (IC) to detect suboptimal sample processing and PCR conditions that may affect the reproducibility between Ct values and bacterial load.

### Laboratory Procedure

The sputum samples were homogenized using a magnetic stirrer, with the speed and duration adjusted based on the viscosity of the sputum. After homogenization, the sample was divided into two sections: 1 ml of the sputum was used for the Xpert MTB/RIF Ultra test and 2 ml for culture and AFB smear microscopy after decontamination. The decontamination procedure involved 6% sodium hydroxide, 2.9% sodium citrate, and N-acetyl-L-cysteine (NALC) as key components. Decontamination of the sputum samples was performed using a final concentration of 1.5% NALC-NaOH solution to concentrate the organisms and enhance detection. The procedure involves a negative control, which is placed between the samples that are being processed to control for cross-contamination.

The Xpert MTB/RIF Ultra assay (Cepheid, Sunnyvale, CA) was performed by adding sample reagent to the sputum sample in a ratio of 2:1 ml of reagent to the sample, followed by Vortexing for a five-minute interval, for 10 minutes. Subsequently, 2 ml of the resulting mixture was loaded into the Xpert MTB/RIF Ultra cartridge and analyzed using the four-module GeneXpert Instrument. The results, available after approximately two hours, and were interpreted using the software version 4.8 (Cepheid, Sunnyvale, CA). Semi-quantitative results were reported as trace, very low, low, medium, high or very high. With trace result is specifically triggered as MTB positive on the presence of IS6110 and IS1081 but the single copy of rpoB probes not detected. For each new lot number of the cartridge, the QC was performed using H37Rv for positive and the decontaminating reagents as a negative control.

For the auramine staining, a slide was prepared using 200 µL of sediment. The slide was heat-fixed for 2 hours at 70°C^10^ then prepared for staining with auramine. The slide was flooded with auramine for 10 minutes, decolorized for 10 seconds, and then stained with potassium permanganate as a counterstain for 2 minutes at room temperature for 5 minutes. Each batch slide being stained included positive and negative control QC slides, specifically BD BBL AFB slides (Becton Dickinson; Sparks, MD, USA). The positive control contains a mixture of unstained *Mycobacterium tuberculosis* H37Ra, which fluoresces bright yellow to yellow-orange upon staining. The negative control contains an unstained mixture of *S. aureus* ATCC 2592 and *K. pneumoniae* ATCC 13883, which do not fluoresce. Finally, the slide was examined using fluorescent microscopy at 20x and 40x magnification, and the organisms were graded following WHO guidelines.^11^ The slides were reviewed by two laboratory technicians, and in case of discrepancies, a third person was involved to clear the query. And the discrepancy between the two tests was addressed by repeating the test by requesting a new sample and performing the requested tests again. If the results were the same, the results were shared with the clinic for further evaluation using other clinical diagnoses before the initiation of the treatment.

### Statistical Analysis

Data analysis was conducted using the R software. Receiver operating characteristic (ROC) curve analysis was employed, and Spearman's coefficient (ρ), along with 95% confidence intervals, was used to evaluate the correlation between Ct values and smear positivity status. The Mann–Whitney test was performed to investigate potential associations between the distribution of Ct values and smear positivity.

### Ethical Considerations

Before commencement, this study received approval from the Local Institute Ethical Review Board with certificate number 2528, ensuring adherence to ethical guidelines for research involving human participants.

## RESULTS

A total of 472 sputum samples suspected of having tuberculosis were received, processed, and tested. Among these, 457 samples had more than 3 ml, which was enough for culture using MGIT, AFB smear microscopy, and Xpert MTB/RIF Ultra testing. The remaining 15 samples, which had less than 2 ml, were prioritized for Xpert MTB/RIF Ultra testing and missed out on AFB smear microscopy and sputum culture.

Table 1 provides an overview of the AFB smear results. Initially, 472 samples were analyzed for TB diagnosis in the study. However, 15 individuals were excluded from the analysis due to missing smear results. Of the 457 smears, microscopy revealed that 38.1% of the participants analysed were negative, 29.8% were classified as Scanty. Among the smear positive cases 17.5% were graded as 1+, 10.5% as 2+, and 4.2% as 3+, reflecting a decreasing trend with increasing bacillary load according to the IUTLD classification, as shown in the Table 1.

**TABLE 1:** Smear Data Results (N=457)

Smear Grade	N (%)
Negative (0)	174 (38.1%)
Scanty	136 (29.8%)
1+	80 (17.5%)
2+	48 (10.5%)
3+	19 (4.2%)

A total of 472 sputum samples were collected and subjected to Xpert MTB/RIF Ultra, testing utilizing the newly endorsed WHO GeneXpert Ultra cartridge.

Table 2 presents the GeneXpert results. Among the 472 samples collected, 4 samples provided invalid results, so they could not be reported as valid results. Among the analysed samples with valid results, 84 (18%) showed a high bacterial detection, while 121 (26%) tested negative via Xpert MTB/RIF Ultra. Notably, participants with low bacterial detection were more prevalent, comprising 141 (30%) cases within this group.

**TABLE 2:** Xpert Ultra totals (N=468)

Xpert Result	N (%)
Not Detected	121 (26%)
Trace	29 (6%)
Very Low	8 (2%)
Low	141 (30%)
Medium	85 (18%)
High	84 (18%)
Very High	0 (0%)

Table 3 presents the Ct value mean and standard deviation (SD) for Xpert Ultra results. The mean Ct values for each reported category were examined, revealing distinct patterns. Distribution of Ct values across bacterial load categories detected by the molecular assay. Bacterial load groups are defined as follows: Trace (Ct = 23.3 ± 6.8, n = 29), Very Low (Ct = 22.5 ± 2.0, n = 8), Low (Ct = 18.5 ± 2.0, n = 141), Medium (Ct = 16.4 ± 0.3, n = 85), and High (Ct = 16.2 ± 0.2, n = 84). Lower Ct values indicate higher concentrations of mycobacterial DNA.

**TABLE 3:** Ct Value Mean and SD for Xpert Ultra Results

Xpert Result (n = 347)	Ct Value, mean ± SD
Trace (n = 29)	23.3±6.8
Very Low (n = 8)	22.5 ± 2.0
Low (n = 141)	18.5 ± 2.0
Medium (n = 85)	16.4 ± 0.3
High (n = 84)	16.2 ± 0.2
Very High (n = 0)	0

Table 4 shows the GeneXpert MTB/RIF Ultra, results for each smear grade. Among the samples included in this study, there is a clear trend indicating that higher semi-quantitative categories on Xpert MTB/RIF Ultra are associated with higher smear microscopy grades. Of the samples with high Xpert results, 72% were smear-positive (1+ or higher), compared to only 10% among those with Trace results. Only 19 individuals had sputum smears categorized as “high” based on smear grading. The remaining patients had smears microscopically reported as “2+,” “1+,” or “scanty.” Interestingly, these patients with smears labelled as “2+,” “1+,” or “scanty” made up 56% of the total cases reported as “high” in Xpert MTB/RIF Ultra, testing. This indicates good agreement between bacillary load detected by Xpert and smear microscopy, although some discordance remains in lower bacillary categories.

**TABLE 4:** Xpert Results Within Each Smear Grade

Xpert ultra result	Smear grade *					
	3+, (%)	2+, (%)	1+, (%)	Scanty, (%)	Negative, (%)	Total
High, n (%)	19	34	19	3	0	75
Medium, n (%)	0	6	40	27	4	77
Low, n (%)	0	4	13	89	33	139
Very Low, n (%)	0	1	0	2	4	7
Trace, n (%)	0	1	3	11	14	29
Total	19	46	75	132	55	327**

* 41 values were missing from smear grade data; total = 427

** 100 values were “Not Detected” on Xpert Ultra; therefore, the total is 327 for the table

**FIGURE 1:**
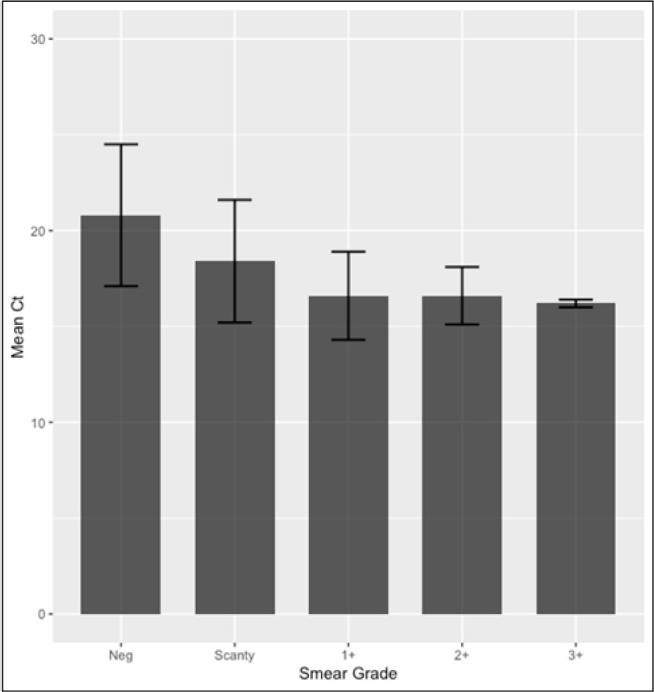
Mean and SD for Ct according to smear grade

Figure 1 displays the mean and standard deviation (SD) for Ct values according to smear grade. The results reveal a negative correlation between the Ct value and smear grading, indicating a decrease in Ct value with higher smear grades. Specifically, cases classified as 3+ smear grade exhibited a lower Ct value, suggesting a higher bacterial burden in sputum samples. Conversely, negative cases displayed Ct values above 20, indicating a notably lower bacterial presence in the sputum.

**FIGURE 2:**
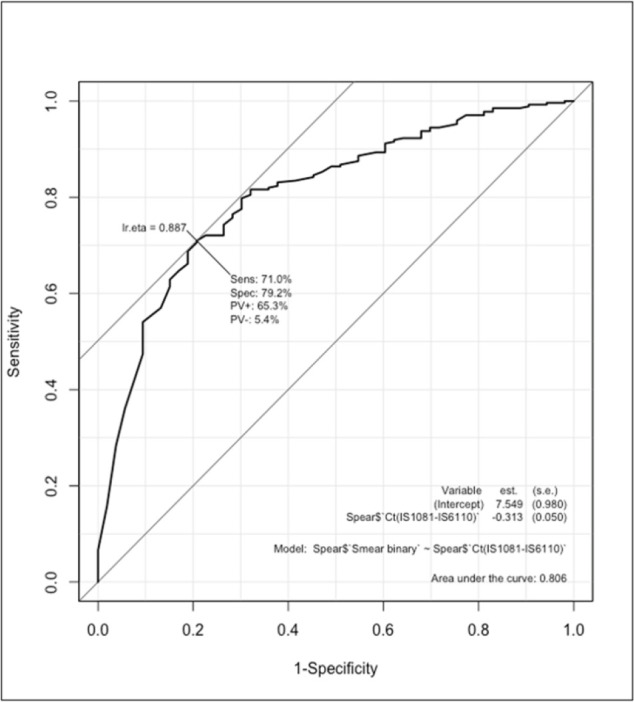
ROC curves with sensitivity and specificity without Ct = 0 values

Figure 2 depicts the ROC curves with sensitivity and specificity for cases without Ct = 0 values. The Receiver Operating Characteristic (ROC) curve analysis conducted between cases with recorded Ct values and smear grades, with Ct values removed where Ct = 0 and considering a smear grade of 0 as negative and everything else as positive, resulted in a cut-off value of 17.45. The analysis yielded a sensitivity of 71% (95% CI 55.2 – 82.7) and specificity of 79.2% (95% CI 64.1 – 89.2), with an area under the curve (AUC) of 0.806.

*Removed Ct values with 0; Considered Smear of 0 negative and everything else positive

Cutoff value = 17.45

Sensitivity = 71% (95% CI 55.2 – 82.7)

Specificity = 79.2% (95% CI 64.1 – 89.2)

AUC = 0.806

**FIGURE 3:**
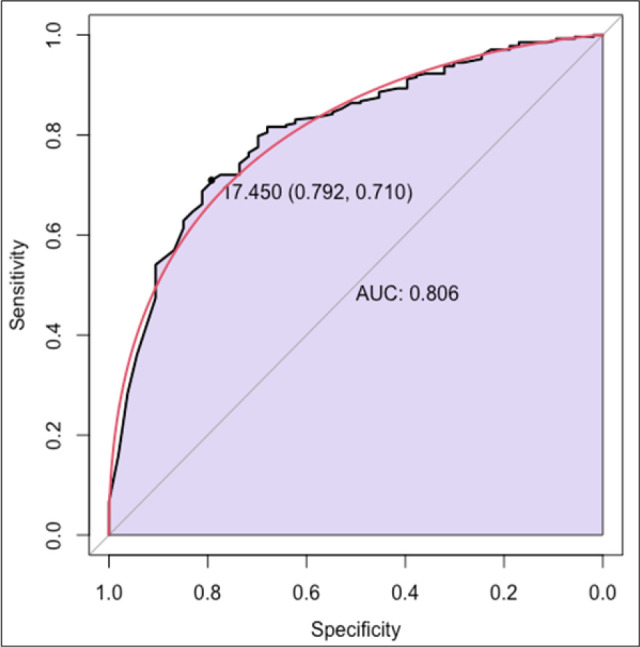
Additional ROC curves with sensitivity and specificity without Ct = 0 values

Spearman's Rank Correlation Rho

S = 9267609, *p*-value < 2.2e-16

rho = −0.6198432

The established Ct cut-off clinically can help identify patients who are more likely to have active disease, guiding decisions on starting treatment or doing more tests.

## DISCUSSION

Both microscopy and Xpert MTB/RIF Ultra, are tests endorsed by WHO at different time points. The era of Xpert MTB/RIF Ultra has improved the diagnosis of TB, and the case detection rate has also increased. In this case, we had the same sputum subjected to both tests, microscopy and Xpert MTB/RIF Ultra, which is the new test endorsed by WHO recently. The two techniques differ in specificity and sensitivity, each offering its advantages and disadvantages. For example, the Xpert MTB/RIF Ultra, is specific and useful for screening with high sensitivity and specificity but performs poorly in treatment monitoring. Smear microscopy is a reliable confirmatory test for MTB and has increased sensitivity with auramine O; it is also a useful tool for treatment monitoring but has low sensitivity for screening.^12–14^ The Xpert MTB/RIF Ultra is very specific with the detection limit of 16 cfu/ml of *M. tuberculosis* spiked into sputum.^15,13^ Smear sensitivity varies greatly based on AFB burden within sputum, with 1000–10,000 CFU/ml required for reliable detection for fluorescence microscopy.^16^ The threshold for detecting bacilli on light microscopy is about 5000−10,000 bacilli/mL, while the infecting dose of *Mycobacterium tuberculosis* is estimated to be fewer than ten organisms.^17^

By performing AFB microscopy, 36% of suspected cases were negative for tuberculosis, highlighting the complexity of diagnosing this disease accurately, necessitating the exploration of more sensitive and specific diagnostic methods. Additionally, 64% of the screened cases were identified as positive, with a small proportion of participants exhibiting a high bacterial load, only 4% according to the IUTLD classification. This raises questions about the severity and distribution of tuberculosis cases within the studied population, of which only 4% are considered highly infectious and have a high transmission rate, while we have seen reports on transmission from AFB-negative cases. ^18^ These findings emphasize the importance of good diagnostic test interpretation and cautious extrapolation of results in a clinical setting. Future studies should aim to address methodological limitations and explore alternative approaches to enhance the accuracy and reliability of tuberculosis diagnosis^17^ using the AFB microscopy and reading technique.

In the continuation of the diagnostic test, we evaluated the performance of the Xpert MTB/RIF, Ultra cartridge in diagnosing tuberculosis. The Xpert MTB/RIF Ultra, could detect 26% as smear-negative from the same group of participants, where microscopy could detect 36% as smear negative. This shows the sensitivity of the Xpert MTB/RIF Ultra test is high in comparison to AFB microscopy, which could increase the detection cases by 10% of the same cohort as MTB positive.^19^ Also, in evaluating the detection of positive cases, the Xpert MTB/RIF Ultra, could identify 74% as positive for MTB and 18% identified with a high bacterial load.^20^ The results from this cohort suggest that the Xpert MTB/RIF Ultra, assay is effective in identifying individuals with active tuberculosis infections. However, the substantial proportion of negative results (26%) highlights the limitations of this diagnostic tool, possibly due to factors such as sample quality or the presence of non-tuberculous mycobacteria.^21^ Interestingly, the prevalence of participants with low bacterial detection, constituting 30% of the cases, underscores the potential significance of subclinical or early-stage tuberculosis infections within the studied population. The clinical implications of discordant results between smear microscopy and Xpert MTB/RIF Ultra are profound not only at the individual patient level but also in shaping treatment decisions, infection control, and broader public health strategies.

Among the patients enrolled in this study, only 19 individuals exhibited sputum smears classified as “high” based on smear grading, indicating a significant portion of the cohort with lower grades. Remarkably, patients with smears categorized as “2+,” “1+,” or “scanty” comprised 56 individuals of the total cases identified as “high” in Xpert MTB/RIF Ultra, testing.^22^ This observation unveils a notable discordance between the traditional smear grading method and the outcomes obtained from Xpert MTB/RIF Ultra, testing in identifying patients with high bacterial loads.

The implications of this incongruity are substantial, potentially affecting the accuracy of tuberculosis diagnosis and subsequent treatment decisions.^23^ Further exploration is imperative to elucidate the underlying factors contributing to this incongruence, whether it be variations in sample collection, differences in detection thresholds between methods, or other factors. Understanding these discrepancies is crucial for refining diagnostic protocols and ensuring more precise identification of patients requiring intensified tuberculosis management strategies.

The analysis of mean Ct values for different reported categories unveils intriguing insights into the distribution of bacterial loads among the studied cases. Notably, cases characterized by high and medium bacterial loads demonstrated a narrow range of Ct values, falling between 16.0 and 16.7. This tight clustering suggests a relatively consistent level of bacterial abundance within these groups, potentially indicating a distinct clinical phenotype or disease severity.^24,25^ Conversely, the remaining groups exhibited a notable divergence in Ct values from each other, implying a broader spectrum of bacterial loads or diverse disease presentations. These findings underscore the heterogeneity inherent in tuberculosis infections and highlight the importance of considering individualized diagnostic and treatment approaches based on bacterial load profiles.^25^ Further exploration of the clinical correlates and prognostic implications associated with varying Ct values across different tuberculosis categories could enhance our understanding of disease progression and inform more targeted intervention strategies.

The findings of our study unveil a significant correlation between the Ct value and smear grading, highlighting a notable trend where lower Ct values correspond to higher smear grades. This observation highlights the relationship between bacterial burden and smear grading, suggesting that as smear grading increases, denoting a greater bacterial concentration, the Ct value tends to decrease. This finding aligns with previous research, which has similarly demonstrated that a lower Ct value correlates with a higher bacterial load in sputum samples.^26,27,28^ Notably, our study reveals cases classified as 3+ smear grade exhibit particularly lower Ct values, signifying a heightened bacterial burden in these samples. Conversely, negative cases, indicating the absence of tuberculosis bacteria, displayed Ct values above 20, suggesting a notably lower bacterial presence in the sputum. This observation is consistent with the notion that higher Ct values correspond to lower bacterial concentrations.^27,25^ Overall, these findings emphasize the utility of Ct values in assessing bacterial burden in tuberculosis cases and highlight the potential of this metric as a diagnostic tool in clinical practice. Clinicians can use lower Ct values indicating high bacillary burden to prioritize rapid treatment initiation, airborne isolation, and contact tracing, while higher Ct values may suggest paucibacillary disease or even non-viable organisms, prompting further diagnostic evaluation before starting therapy. This approach helps refine treatment decisions and reduces unnecessary use of multidrug-resistant TB regimens, especially in cases where high Ct values are associated with false-positive rifampicin resistance. From a cost-effectiveness standpoint, integrating Ct values can optimize resource allocation by minimizing inappropriate isolation, unnecessary treatment, and redundant testing.

The findings from the ROC curve analysis highlight the potential of Ct values in predicting smear grades in cases of certain diseases. With a sensitivity of 71.0% and specificity of 79.2%, these results underscore the utility of Ct values as a predictive biomarker for diagnosis and assessing disease progression. Such predictive capabilities hold significant clinical implications, particularly in the treatment monitoring of infectious diseases, where early and accurate diagnosis, effective management, and treatment decisions are crucial.^24^ These results align with prior research emphasizing the value of Ct values in disease prognosis and risk stratification.^29^ However, it's essential to acknowledge the limitations of this study, including potential confounding factors and the need for further validation in larger cohorts or diverse populations. Nonetheless, the demonstrated sensitivity and specificity of Ct values in predicting smear grades suggest promising avenues for incorporating this parameter into clinical practice, potentially enhancing diagnostic accuracy and informing personalized treatment strategies.

## CONCLUSION

In conclusion, this study highlights the persistent challenge of diagnosing and managing TB, a global health threat despite significant efforts and advancements in treatment regimens and diagnostic techniques. The findings underscore the importance of accurate and sensitive diagnostic methods in combating TB, particularly given the disease's high mortality rate in the absence of proper medical intervention.

The evaluation of the Xpert MTB/RIF Ultra test and smear microscopy reveals insights into the correlation between bacterial load and disease severity, emphasizing the potential of molecular diagnostic tools in providing rapid and precise assessments of TB infections. However, discrepancies between traditional smear grading methods and molecular test outcomes underscore the complexity of TB diagnosis and the need for further research to refine diagnostic protocols.

The study's findings regarding the correlation between Ct values and smear grading demonstrate the potential utility of Ct values as predictive biomarkers for assessing disease severity and progression, offering promising prospects for enhancing TB diagnosis and treatment strategies.

Overall, the study contributes valuable insights into the ongoing efforts to combat TB and underscores the importance of continued research and innovation in tuberculosis diagnostics and management.

### Limitations

The study presented notable limitations that warrant consideration. Firstly, the research primarily focused on a specific geographical region, the Kilimanjaro region, which may limit the generalizability of the findings to broader populations with diverse demographic and epidemiological characteristics. The exclusion of 13 samples from the smear microscopy analysis due to insufficient sample volume, while still performing and analyzing Xpert MTB/RIF Ultra, testing on these same samples, represents a methodological limitation of the study. This discrepancy introduces the potential for selection bias, as the absence of smear results for these cases may impact the accuracy of correlation analyses and overall interpretability of findings. While the enrolment of 472 participants initially provided a robust sample size, the loss of these individuals could impact the generalizability of the results.

Additionally, the study's reliance on sputum samples for diagnostic evaluation introduces potential biases related to sample quality and adequacy, which could impact the accuracy of test results. Moreover, the study acknowledged discrepancies between traditional smear grading methods and GeneXpert MTB/RIF Ultra, results in the identification of patients with high bacterial loads, indicating potential limitations in existing diagnostic protocols.

Furthermore, while the study highlighted the correlation between Ct values and smear grading, it is important to recognize the variability in Ct values across different tuberculosis categories, suggesting the need for further investigation into the clinical correlates and prognostic implications associated with varying Ct values. The absence of clinical outcome data to validate the utility of Ct values represents a key limitation of this study. While Ct values appeared to be a critical finding, their correlation with treatment outcomes was not assessed. As a result, the prognostic or clinical significance of Ct values remains unverified within this context, limiting the applicability of the findings to patient management or treatment response evaluation.

Lastly, while the ROC curve analysis demonstrated promising sensitivity and specificity of Ct values in predicting smear grades, the study acknowledged the need for validation in larger cohorts or diverse populations to ensure the robustness and reliability of these predictive biomarkers in clinical practice, and also include the cost-effective analysis. The predictive utility of GeneXpert Ct values can enhance clinical decision-making by serving as proxies for bacillary load; however, their interpretation is influenced by confounding factors such as HIV co-infection, poor sputum quality, prior TB history, and laboratory variability, all of which can lead to discrepancies in diagnostic performance. To ensure accurate and cost-effective integration into clinical workflows, Ct values must be considered alongside clinical context, sample integrity, and health system constraints

Overall, while the study provided valuable insights into the diagnostic utility of Ct values and highlighted potential areas for improvement in tuberculosis diagnosis, addressing these limitations will be essential for advancing our understanding and management of this global health threat.

### Recommendation

Based on the comprehensive study conducted on the diagnosis of TB utilizing both smear microscopy and the Xpert MTB/RIF Ultra test, it is evident that advancements in diagnostic techniques are pivotal for improving TB management and control. The study's findings underscore the significance of the Xpert MTB/RIF Ultra test in accurately detecting *Mycobacterium tuberculosis* DNA in sputum samples, providing rapid and reliable results crucial for timely intervention. Notably, the correlation observed between the Ct value obtained from the Xpert MTB/RIF Ultra test and smear grading highlights the potential of Ct values as predictive indicators of disease severity and progression.

The study's meticulous methodology, including a prospective cross-sectional design and comprehensive laboratory procedures, ensures robust data collection and analysis. The ethical considerations taken into account, with approval from both local and national ethical review boards, demonstrate a commitment to maintaining ethical standards in research.

The results obtained from the study reveal intriguing insights into TB diagnosis, particularly regarding the discrepancies observed between smear microscopy and Xpert MTB/RIF Ultra, testing in identifying patients with high bacterial loads. Such findings underscore the need for continued research to refine diagnostic protocols and ensure more accurate TB diagnosis and subsequent treatment decisions.

Furthermore, the analysis of mean Ct values across different TB categories sheds light on the heterogeneity of TB infections and highlights the potential utility of Ct values as diagnostic biomarkers. The ROC curve analysis further emphasizes the predictive capabilities of Ct values in assessing disease severity, with promising sensitivity and specificity observed.

Overall, this study's findings contribute significantly to the ongoing efforts to combat TB globally. By elucidating the strengths and limitations of current diagnostic techniques and highlighting areas for further research and improvement, this study paves the way for enhanced TB diagnosis, management, and control strategies.
